# Proton magnetic resonance spectroscopy detects cerebral metabolic derangement in a mouse model of brain coenzyme a deficiency

**DOI:** 10.1186/s12967-022-03304-y

**Published:** 2022-02-23

**Authors:** Yanan Li, Jeffrey Steinberg, Zane Coleman, Shubo Wang, Chitra Subramanian, Yimei Li, Zoltan Patay, Walter Akers, Charles O. Rock, Suzanne Jackowski, Puneet Bagga

**Affiliations:** 1 Department of Diagnostic Imaging, St. Jude Children’s Research Hospital, Memphis, TN USA; 2 Center for In Vivo Imaging and Therapeutics (CIVIT), St. Jude Children’s Research Hospital, Memphis, TN USA; 3 Department of Infectious Diseases, St. Jude Children’s Research Hospital, Memphis, TN USA; 4 Department of Biostatistics, St. Jude Children’s Research Hospital, Memphis, TN USA

**Keywords:** Pantothenate kinase, Coenzyme A, Neurodegeneration, Pantothenate kinase-associated neurodegeneration, ^1^H magnetic resonance spectroscopy, Metabolites, Therapeutics

## Abstract

**Background:**

Pantothenate kinase (PANK) is the first and rate-controlling enzymatic step in the only pathway for cellular coenzyme A (CoA) biosynthesis. PANK-associated neurodegeneration (PKAN), formerly known as Hallervorden–Spatz disease, is a rare, life-threatening neurologic disorder that affects the CNS and arises from mutations in the human *PANK2* gene. Pantazines, a class of small molecules containing the pantazine moiety, yield promising therapeutic effects in an animal model of brain CoA deficiency. A reliable technique to identify the neurometabolic effects of PANK dysfunction and to monitor therapeutic responses is needed.

**Methods:**

We applied ^1^H magnetic resonance spectroscopy as a noninvasive technique to evaluate the therapeutic effects of the newly developed Pantazine BBP-671.

**Results:**

^1^H MRS reliably quantified changes in cerebral metabolites, including glutamate/glutamine, lactate, and N-acetyl aspartate in a neuronal *Pank1 and Pank2* double-knockout (*SynCre*^+^ *Pank1,2* dKO) mouse model of brain CoA deficiency. The neuronal *SynCre*^+^ *Pank1,2* dKO mice had distinct decreases in Glx/tCr, NAA/tCr, and lactate/tCr ratios compared to the wildtype matched control mice that increased in response to BBP-671 treatment.

**Conclusions:**

BBP-671 treatment completely restored glutamate/glutamine levels in the brains of the mouse model, suggesting that these metabolites are promising clinically translatable biomarkers for future therapeutic trials.

**Supplementary Information:**

The online version contains supplementary material available at 10.1186/s12967-022-03304-y.

## Background

**Fig. 1:**
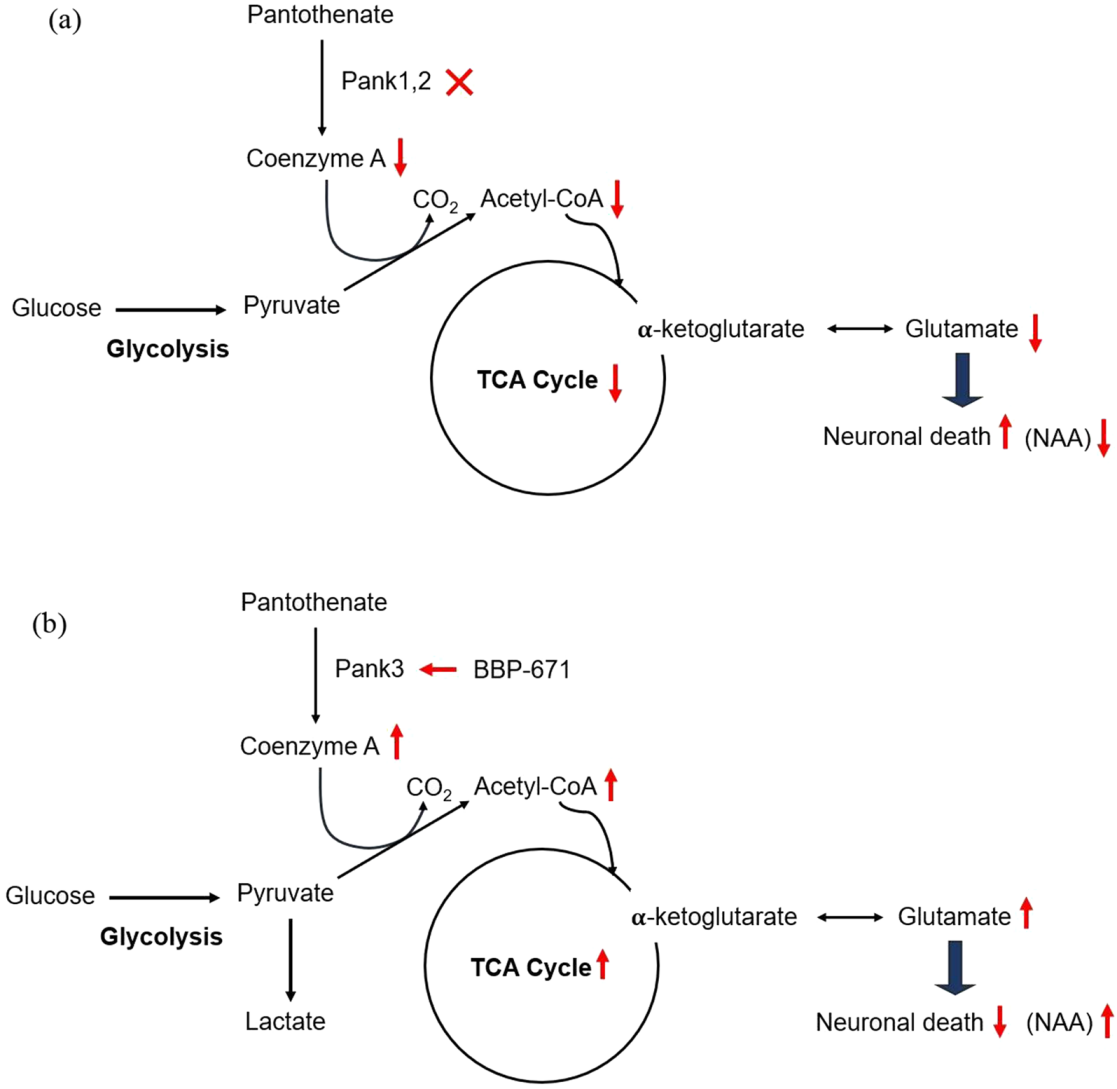
**a** Schematic description of CoA loss due to *Pank1,2* deletion in neurons leading to decreased TCA cycling and lower neuronal glutamate and NAA. **b** Schematic decription of CoA recovery after treated with BBP-671 therapeutic

Pantothenate kinase (PanK) is the first and rate-controlling step in the only pathway for coenzyme A (CoA) biosynthesis [1]. CoA is essential for hundreds of metabolic reactions including the tricarboxylic acid (TCA) cycle, fatty acid oxidation and synthesis, amino acid metabolism, and neurotransmitter synthesis [1, 2]. A rare, life-threatening neurological disorder known as pantothenate kinase-associated neurodegeneration (PKAN) arises from mutations in the human *PANK2* gene leading to a prominent extrapyramidal movement disorder and a characteristic deposition of iron in the basal ganglia [3]. CoA deficiency is thought to be the cause of the movement disorder and neurodegeneration in PKAN [4], but probes are not available to monitor CoA levels in the brain. Figure 1a depicts the effects of loss of CoA synthesis due to disruption of murine *Pank1* and *Pank2* genes in neurons. Pyruvate produced from glycolysis requires CoA to form acetyl-CoA, a substrate for the mitochondrial TCA cycle, and CoA limitation can disrupt TCA cycling [5] which, in turn, affects glutamate metabolism thereby causing cell death [6, 7] (Fig. 1a). Recent reviews discuss the various molecular consequences of CoA insufficiency in preclinical PKAN models [8, 9]. There are no disease-modifying treatments for PKAN, and therapeutics are desperately needed. A new potential PKAN therapeutic approach uses Pantazine small molecules that activate the PanK isoforms and thereby stimulate CoA production [10]. Pantazine PZ-2891 was previously reported to restore cerebral CoA levels in the *SynCre*^+^ *Pank1,2* neuronal dKO mice [10]. In this study, we used proton magnetic resonance spectroscopy (^1^H MRS) to evaluate the brain metabolic derangements that are associated with neuronal CoA deficiency in this mouse model, with and without treatment with a newly developed Pantazine, BBP-671. ^1^H MRS is particularly advantageous for this study because it permits the noninvasive examination of the most abundant cerebral metabolites in vivo [11]*.* This Pantazine allosterically activates the alternate PanK3 isoform that is expressed in murine neurons and a proposed mechanism for the neurometabolic effect of CoA restoration by BBP-671 is shown in Fig. 1b.

**Fig. 2:**
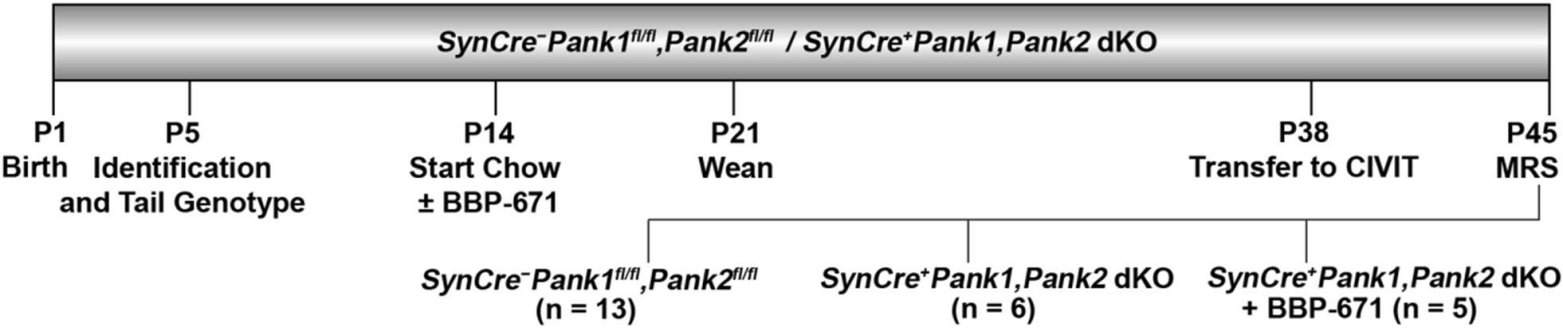
Timeline of events extended from birth at postnatal day 1 (P1) through P45 when animals were anesthetized and midbrains were evaluated by magnetic resonance spectroscopy (MRS). Individual animals were identified and tail genotyping was performed at P5. Animals were assigned to three groups: *SynCre*^+^*Pank1,2* dKO ± BBP-671 and *SynCre*^−^*Pank1*^*fl/fl*^*,Pank2*^*fl/fl*^ matched wildtype controls, and were housed with control nursing dams at P14. Animals were maintained on the purified chow diet ± 75 ppm BBP-671 starting at P14 through P45, and weaned at P21. Mice were transferred to the Center for In Vivo Imaging and Therapeutics (CIVIT) at approximately P38 and MRS was performed at approximately P45. Numbers of mice in each group are indicated in parentheses

^1^H MRS is a powerful and non-invasive technique which can provide information about neurometabolites in vivo [11, 12]. ^1^H MRS can identify biomarkers and be applied to evaluate clinical neurodegeneration and therapeutic strategies [13, 14]. In the limited clinical studies demonstrating the application of ^1^H MRS in studying PKAN, a reduced N-acetyl aspartate (NAA) level was commonly observed [15–18]. This is the first report using ^1^H MRS for monitoring PKAN therapeutics in a mouse model of the disease. In this study, we treated *SynCre*^+^*Pank1,2* neuronal dKO mice with BBP-671 and used ^1^H MRS to identify the prominent neurochemical metabolites and evaluate treatment response (Fig. 2). We found that the cerebral metabolite levels, including glutamate + glutamine (Glx), NAA and lactate, were altered in the mid-brain of the animal model. Based on our observations, we show that Glx levels may be an indicator of restored CoA levels in the response to BBP-671 treatment.

## Methods

### Animal care and use

All procedures were reviewed and approved by the St. Jude Children’s Research Hospital Institutional Animal Care and Use Committee. The *SynCre*^+^*Pank1,Pank2* neuronal dKO mice were generated as described in Sharma et al. [10]. The mice were maintained at room temperature, humidity 50 ± 10% and a 14/10-h light/dark cycle. Animals were maintained on a purified chow diet (Envigo TD.170542) ± 75 ppm BBP-671. Mice were put on study on a rolling basis as they emerged from the breeding program and were randomized into the treatment arms at postnatal day 14 (P14). Water was supplied ad libitum. *SynCre*^+^*Pank1,2* neuronal dKO (n = 5) received BBP-671 fortified chow for approximately 31 days. The steady state levels of BBP-671 in plasma (1 mM) from mice on 75 ppm BBP-671 chow were determined 2–4 h after cessation of the dark cycle by mass spectrometry analyses as described previously [10] and found to be consistent within the treatment group. *SynCre*^+^*Pank1,2* neuronal dKO (n = 6) and *SynCre*^−^*Pank1*^*fl/fl*^*,Pank2*^*fl/fl*^ wild type controls (n = 13) received matched chow without BBP-671 for the same length of time. MRS was performed on mice at an age of 6–7 weeks (P42–P49). In a separate study, the *SynCre*^+^*Pank1,Pank2* neuronal dKO mice treated with BBP-671 had improved lifespan, weight gain and locomotion similar to previous results using PZ-2891 [10]. Neuron-specific deletion of *Pank1* and *Pank2* was confirmed by PCR genotyping of brain and liver, and BBP-671 levels in brains were confirmed postmortem as described previously [10].

### ^1^H magnetic resonance spectroscopy in mice

MRI/MRS studies were performed on a Bruker Clinscan 7T magnetic resonance imaging (MRI) scanner (Bruker BioSpin MRI GmbH, Ettlingen, Germany). Mice were anesthetized using isoflurane mixed with oxygen (1–2%) and the respiration rate was monitored. The total scan time was 21 min. MRI was acquired with a mouse brain surface receive coil positioned over the mouse head and placed inside a 72 mm transmit/receive coil. After the localizer, a T_2_-weighted turbo spin echo sequence was performed in the coronal (TR/TE = 2290/41 ms, matrix size = 192 × 256, slice thickness 0.5 mm, number of slices = 14) and axial (TR/TE = 3841/50 ms, matrix size = 192 × 144, slice thickness 0.4 mm, number of slices = 42) orientations. The T_2_-weighted scans were used to position a 3.5 × 4.5 × 2.0 mm^3^ voxel for spectroscopy in the midbrain to cover thalamus and hippocampus. A ^1^H MR spectrum was generated with that voxel using a PRESS sequence (repetition time/echo time = 3000/11 ms, averages = 128, data length = 2048, spectral width = 2900 Hz). Voxel positioning is displayed in Additional file 1: Fig. S2.

### ^1^H MRS data processing

Metabolite to tCr ratios measured by in vivo ^1^H MRS were quantified using LCModel software (v.6.3), a widely applied MRS analysis tool that employs a least-squares-based prior-knowledge fitting program [19]. LCModel applied a 7 T spin echo (TE = 11 ms) basis set incorporating the following resonances: alanine (Ala), aspartic acid (Asp), creatine (Cre), phosphocreatine, g-amino butyric acid (GABA), glucose, glutamine (Gln), glutamate (Glu), glycerophosphocholine, phosphocholine, glutathione, myo-inositol (m-Ins), N-acetyl aspartate (NAA), NAA + Glu, sycllo-inositol and taurine, with lipid resonances at 0.9, 1.3 and 2.0 ppm and macromolecule resonances at 0.9, 1.2, 1.4, 1.7 and 2.0 ppm. The unsuppressed water signal was not acquired, therefore metabolite concentrations are reported relative to total creatine (tCr) as applied by others [20–23].

### Statistics

Mean and standard deviation were calculated for MRS parameters in each mouse group. Wilcoxon Rank sum tests were used to test whether MRS parameters are different between wild type, *SynCre*^+^*Pank1,Pank2* dKO or *SynCre*^+^*Pank1,Pank2* dKO + BBP-671 groups. Bonferroni correction was done for the nine comparisons, and thus a *p*-value < 0.05 was deemed to be statistically significant. All the analyses were done using ‘R’ 4.0.2.

## Results

**Fig. 3:**
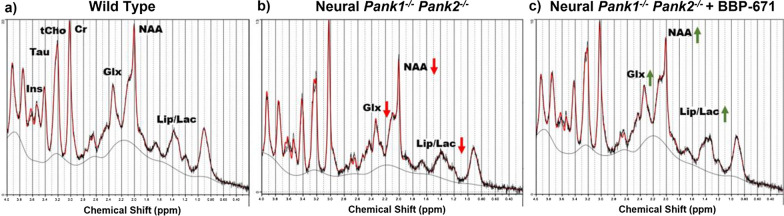
Representative ^1^H MR spectra from three groups **a** Wild Type, **b** Neural *Pank1,2* dKO, and **c** Neural *Pank1,2* dKO mice treated with BBP-671

**Fig. 4:**
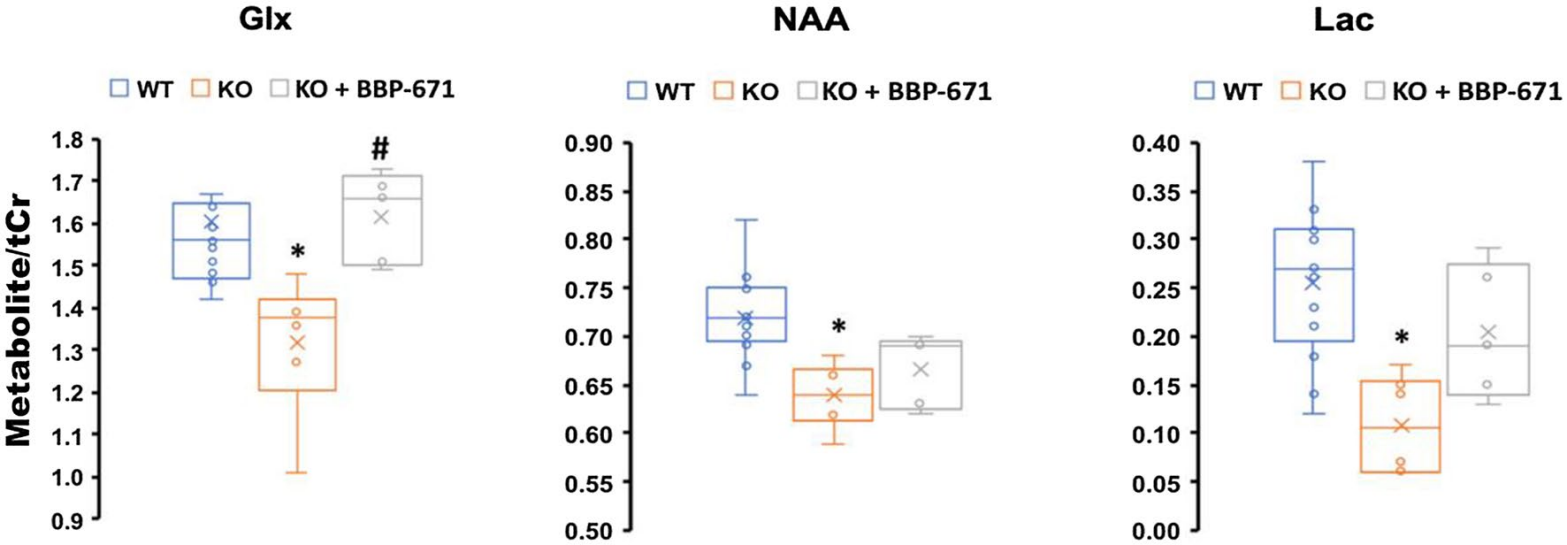
Metabolite to total creatine ratios of glutamate + glutamine (Glx), N-acetyl aspartate (NAA) and lactate (Lac); *p < 0.05 (WT vs KO); ^#^p < 0.05 (KO vs. KO + BBP-671). The box in the box and whisker plot represents the first and third quartiles. The line within the box represents the median while the ‘x’ within the box represents the mean

The *SynCre*^+^*Pank1,2* neuronal dKO mice show clear physiological symptoms of CoA deficiency such as reduced growth rate and impaired locomotor activity [10]. Figure 3 represents the ^1^H MRS spectra acquired from the midbrain of representative mice in each group. There is a clear reduction in the Glx/tCr, NAA/tCr and lactate/tCr ratios in the midbrains of the *SynCre*^+^*Pank1,2* neuronal dKO mice compared to WT (Wildtype). Glx is the summed group of Glu and Gln, where Glu is the most abundant excitatory neurotransmitter in brain and Gln is the main precursor for Glu [13, 24]. Reduction of Glx points toward impaired excitatory neuronal metabolism in the dKO mice and Glx fully recovered following treatment with BBP-671 (Fig. 4, Additional file 1: Table S1). Reduction of NAA indicated loss of neuronal integrity and function, and the NAA/tCr ratio trended toward improvement but was not significant following BBP-671 treatment. Reduction of NAA in the globus pallidus is consistently reported in patients with PKAN [15–18]. The lactate/tCr ratio was lower in the dKO mice representing the net balance between lactate production and consumption [13]. Reduced cerebral glycolysis and/or enhanced lactate oxidation to pyruvate to maintain the redox state in the neurons is suggested from the data. The lactate/tCr ratio trended toward improvement with treatment but without statistical significance. The metabolic recovery following therapy, together with a strong signal strength in the ^1^H MRS points to Glx as a promising biomarker. NAA and lactate are also candidate markers but require further study. While no changes were observed, additional metabolites—inositol, choline, and taurine—are shown in Additional file 1: Fig. S1.

## Discussion

The treatment response to BBP-671 was associated with recovery of cerebral Glx. BBP-671 treatment activates the PANK3 isoform to increase CoA production that, in turn, has a direct role on TCA cycle metabolism (Fig. 1) [5] and hence Glx production. In contrast to our findings, a study performed on 3 human PKAN patients reported an increase of Glx in the white matter [25]. However, the clinical study was performed in patients with chronic disease progression and potential involvement of multiple brain cell types while this preclinical study was performed in a mouse model with rapidly progressing disease that is specific to neurons. Although neurons have been identified as a focal target of disease in PKAN [26], the role of glial cells in disease progression, for example, is not understood. A clinical MRS study reported elevated myo-inositol (m-Ins) levels in white matter due to gliosis and glial proliferation in patients with PKAN [16]. However, we did not find any significant changes in the m-Ins levels in the *SynCre*^+^*Pank1,2* neuronal dKO mice.

The *SynCre*^+^*Pank1,2* neuronal dKO mouse model represents only selected molecular effects of PKAN, and the study has limitations. Alternative mouse models were not appropriate for evaluation of cerebral MRS either due to the lack of disease-related movement dysfunction and normal lifespan [27] or early postnatal death [4, 28]. Analysis of human PKAN brains identified gene expression signatures that indicated neurons as a focal target of disease [26] and development of the *SynCre*^+^*Pank1,2* neuronal dKO mouse model resulted in a longer-lived animal with consistent and measurable movement dysfunction [13] that was tractable for MRS. Previous studies reported white matter pathology in the patients of PKAN [29]. The previous ^1^H MRS studies in human patients were often performed in white matter [18], which is not easily detected in the mouse model since the mouse brain consists of less than 12% white matter [30], as compared to 43% white matter in humans [31]. Mouse models are recognized as not effective for studying white matter pathologies [32]. In addition, there was no observable iron accumulation in the model, in contrast with the iron accumulation often observed in the basal ganglia of PKAN patients. This study may be limited to detection of metabolic changes in the gray matter in the *Pank1,2* neuronal dKO mice.

PKAN is a life-threatening neurological disorder that can be caused by a variety of *PANK2* mutations that affect protein expression, enzyme activity and/or stability [33, 34], resulting in loss or reduction of kinase activity [33]. PKAN diagnosis is based on characteristics of the movement dysfunction, exon sequencing and MRI evidence for iron accumulation in the basal ganglia [35] with a characteristic “eye-of-the-tiger” pattern in T_2_-weighted images [25, 36]. Our study showed no qualitative differences among the three groups in the T_2_-weighted images. There are currently no approved clinical therapies for this genetic disorder [37]. Progress with newly developing PKAN therapeutics has been made by evaluating mouse models with deactivated *Pank* genes in which brain CoA biosynthesis has been disrupted [9, 10, 38]. One of the current challenges is to reliably identify molecular events associated with PKAN dysfunction in addition to monitoring therapeutic responses [39]. Thus, a technique which can quantitatively, non-invasively, and reproducibly analyze neurometabolism is needed and has potential value.

## Conclusion

In summary, we have successfully applied ^1^H MRS to investigate neuronal chemical alterations in a mouse model of brain CoA deficiency, thought to be an underlying cause of PKAN. The effects of a potential PKAN therapeutic were evaluated by comparing the cerebral metabolic derangements among three mouse groups, where reductions of important metabolites (Glx, NAA, and Lactate) were observed in the model of brain CoA deficiency and recoveries of the same metabolites were found following treatment with a newly developed Pantazine, BBP-671. The most promising biomarker for this potential PKAN therapeutic was the recovery of the Glx/tCr ratio. This study shows that ^1^H MRS can be a powerful tool in evaluating therapeutics for metabolic responses of neurological disorders.

## Supplementary Information

**Additional file 1:** **Table S1.** Mean values and standard deviations for Fig. 4. **Table S2.** The *p* values for Fig. 4. **Figure S1.** Metabolite to total creatine ratio for m-inositol, total choline, and taurine. KO, untreated *Pank1/2* neuronal dKO mice; KO + BBP-671, BBP-671–treated *Pank1/2* neuronal dKO mice; WT, wild-type. **Figure S2.** Voxel Positioning for Fig. 2 shown in a wild-type mouse. The viewpoints are A) Horizontal; B) Sagittal; C) Coronal.
