# Supplementary material for: Proton magnetic resonance spectroscopy detects cerebral metabolic derangement in a mouse model of brain coenzyme a deficiency
Source: J Transl Med. 2022 Feb 23;20:103. doi: 10.1186/s12967-022-03304-y (PMC8867880; doi:10.1186/s12967-022-03304-y)
Supplement: Supplementary file 1 — Additional file 1: Table S1. Mean values and standard deviations for Fig. 4. Table S2. The p values for Fig. 4. Figure S1. Metabolite to total creatine ratio for m-inositol, total choline, and taurine. KO, untreated Pank1/2 neuronal dKO mice; KO + BBP-671, BBP-671–treated Pank1/2 neuronal dKO mice; WT, wild-type. Figure S2. Voxel Positioning for Fig. 2 shown in a wild-type mouse. The viewpoints are A) Horizontal; B) Sagittal; C) Coronal. [file 12967_2022_3304_MOESM1_ESM.docx]

**Additional file 1**

**Table S1.** Mean values and standard deviations for Figure 4.

| **Metabolite/tCr** | **WT** | **KO** | **KO + BBP-671** |
| --- | --- | --- | --- |
| Glx | 1.60 ± 0.21 | 1.32 ± 0.17 | 1.62 ± 0.11 |
| NAA | 0.72 ± 0.04 | 0.64 ± 0.03 | 0.67 ± 0.04 |
| Lac | 0.25 ± 0.08 | 0.11 ± 0.05 | 0.20 ± 0.07 |

Glx, glutamate/glutamine; KO, untreated *Pank1/2* neuronal dKO mice; KO + BBP-671, BBP-671–treated *Pank1/2* neuronal dKO mice; Lac, lactate; NAA, N-acetyl aspartate; tCr, total creatine; WT, wild-type.

**Table S2.** The *p* values for Figure 4.

| **Comparison groups** | **Glx/tCr** | **NAA/tCr** | **Lac/tCr** |
| --- | --- | --- | --- |
| WT vs. *Pank1/2* KO | 0.02 | 0.02 | 0.03 |
| WT vs. KO + BBP-671 | 2.70 | 0.20 | 1.95 |
| *Pank1/2* KO vs. KO + BBP-671 | 0.04 | 1.49 | 0.60 |

Glx, glutamate/glutamine; KO, untreated *Pank1/2* neuronal dKO mice; KO + BBP-671, BBP-671–treated *Pank1/2* neuronal dKO mice; Lac, lactate; NAA, N-acetyl aspartate; tCr, total creatine; WT, wild-type.

**Figure S1.** Metabolite to total creatine ratio for m-inositol, total choline, and taurine. KO, untreated *Pank1/2* neuronal dKO mice; KO + BBP-671, BBP-671–treated *Pank1/2* neuronal dKO mice; WT, wild-type.

**Figure S2.** Voxel Positioning for Figure 2 shown in a wild-type mouse. The viewpoints are A) Horizontal; B) Sagittal; C) Coronal.

**
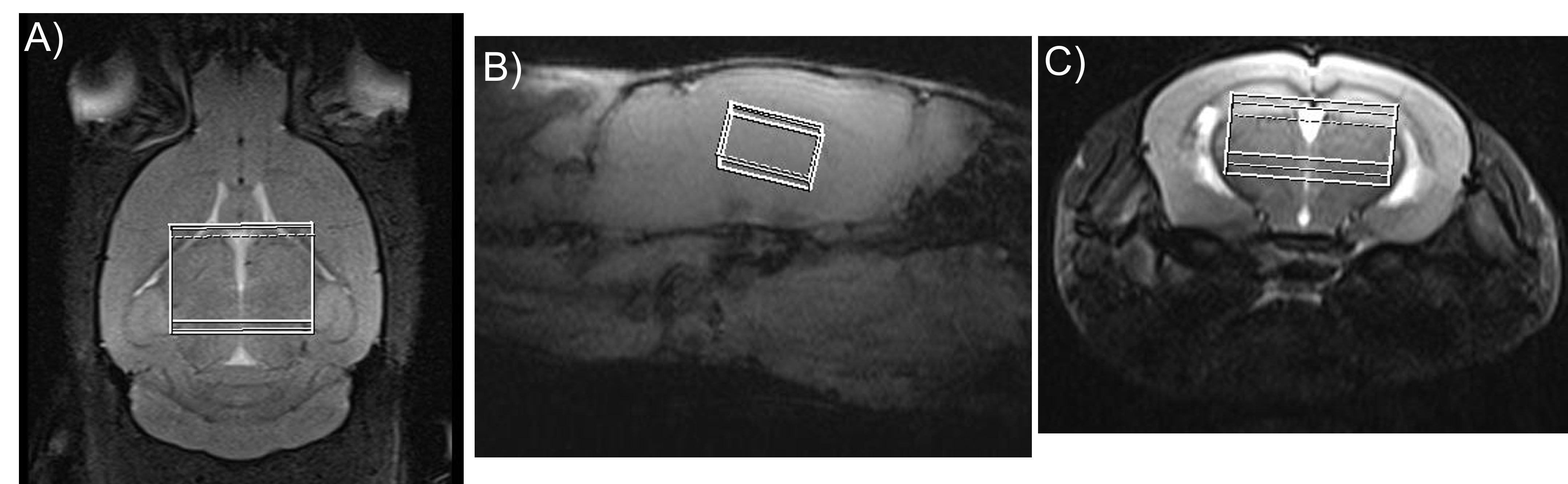
**
